# Online availability of antibiotics from within the UK: shifting patterns from 2016 to 2023

**DOI:** 10.1093/jac/dkae341

**Published:** 2024-10-22

**Authors:** Sara Elizabeth Boyd, Nina Zhu, Laura Whitney, Rohan Surya, Alison Helen Holmes, Raheelah Ahmad

**Affiliations:** David Price Evans Global Health and Infectious Disease Research Group, University of Liverpool, Institute of Systems, Molecular & Integrative Biology, William Henry Duncan Building, 6 West Derby Street, Liverpool L7 8TX, UK; National Institute for Health Research Health Protection Research Unit in Healthcare Associated Infections and Antimicrobial Resistance, Imperial College London, Du Cane Road, London W12 0HS, UK; Infection Clinical Academic Group, St. George’s Hospital NHS Foundation Trust, Blackshaw Road, London SW17 0QT, UK; National Institute for Health Research Health Protection Research Unit in Healthcare Associated Infections and Antimicrobial Resistance, Imperial College London, Du Cane Road, London W12 0HS, UK; Pharmacy Team, Medical Directorate, NHS England, London Region, Wellington House, London SE1 8UG, UK; Health Services Research & Management, School of Health and Medical Sciences, City St. George’s University of London, London, UK; David Price Evans Global Health and Infectious Disease Research Group, University of Liverpool, Institute of Systems, Molecular & Integrative Biology, William Henry Duncan Building, 6 West Derby Street, Liverpool L7 8TX, UK; National Institute for Health Research Health Protection Research Unit in Healthcare Associated Infections and Antimicrobial Resistance, Imperial College London, Du Cane Road, London W12 0HS, UK; National Institute for Health Research Health Protection Research Unit in Healthcare Associated Infections and Antimicrobial Resistance, Imperial College London, Du Cane Road, London W12 0HS, UK; Health Services Research & Management, School of Health and Medical Sciences, City St. George’s University of London, London, UK

## Abstract

**Background:**

We previously reported a cross-sectional analysis of online pharmacy practices and processes. Since then, the demand for and context of online healthcare has changed. However, the current state of access to and usage of antibiotics obtained online remains poorly understood.

**Objectives:**

This study aimed to: (i) determine the legality of online pharmacies selling antibiotics in the UK; (ii) describe processes for obtaining antibiotics online; (iii) identify antimicrobial stewardship (AMS) and patient safety issues; and (iv) compare data with those obtained in 2016 to understand changes in context, and set priorities for targeted research in antibiotic access and usage.

**Methods:**

Searches for ‘buy antibiotics online’ were conducted using ‘Google’ and ‘Yahoo’. The first 10 websites with unique URL addresses for each were reviewed. Analyses were conducted on evidence of pharmacy registration, prescription requirement, whether choice was ‘prescriber-driven’ or ‘consumer-driven’, and whether information was required (allergies, comorbidities, pregnancy) or given (adverse effects) prior to purchase.

**Results:**

Twenty unique URL addresses were analysed. Those evidencing UK location (*n* = 20; 100%) required a prescription and were appropriately registered. For 11 (55%) online pharmacies, decisions were initially consumer-driven for antibiotic choice, but not for dose or duration; contrasting with 2016 when for most (*n* = 16; 80%), decisions were consumer-driven for antibiotic choice, dose and quantity.

**Conclusions:**

Variation continues to exist in relation to antibiotic practices online. We make several key recommendations for lawmakers and stakeholders. Targeted research, improved public engagement, professional education and new best practice guidelines are urgently needed for online UK antibiotic suppliers.

## Introduction

Critical to antimicrobial stewardship (AMS) is the healthcare systemwide approach to promoting and monitoring the judicious use of antimicrobials, which include antibiotics, with the overarching aim to optimize therapy and to preserve their effectiveness.^1^ AMS remains a priority within the UK,^2^ and globally,^3^ with antimicrobial resistance (AMR) continuing to threaten all aspects of healthcare with enormous social and economic impacts.^4,5^ In 2015, the WHO published a global action plan (GAP) for tackling AMR, with specific objectives to ensure medicines are safe, effective and accessible to those who need them, placing particular emphasis on those obtained through internet sales.^3^ In the same year, UN member states committed to developing national action plans (NAPs) to address AMR.

In 2016, we reported that AMS and patient safety strategies were threatened in the UK due to antibiotics being available to purchase online through legal registered pharmacy platforms and illegal websites.^6^ All online pharmacies operating illegally were reported to the Medicines and Healthcare Regulatory Agency (MHRA), who advised they were working with stakeholders to improve patient safety and antibiotic responsibility in this area.^7^ Those that were identified ceased operations. Our findings generated significant media coverage, in addition to formal responses expressing concern from the Royal Pharmaceutical Society (RPS), the Royal College of General Practitioners (RCGP) and the British Dental Association (BDA).^8,9^ The General Medical Council (GMC),^10^ RPS^11^ and the General Pharmaceutical Council (GPhC)^12^ have since published updated guidance for remote prescribing, whilst NHS England has produced an implementation toolkit for ‘Using Online Consultations in Primary Care’.^13^

**Figure 1.**
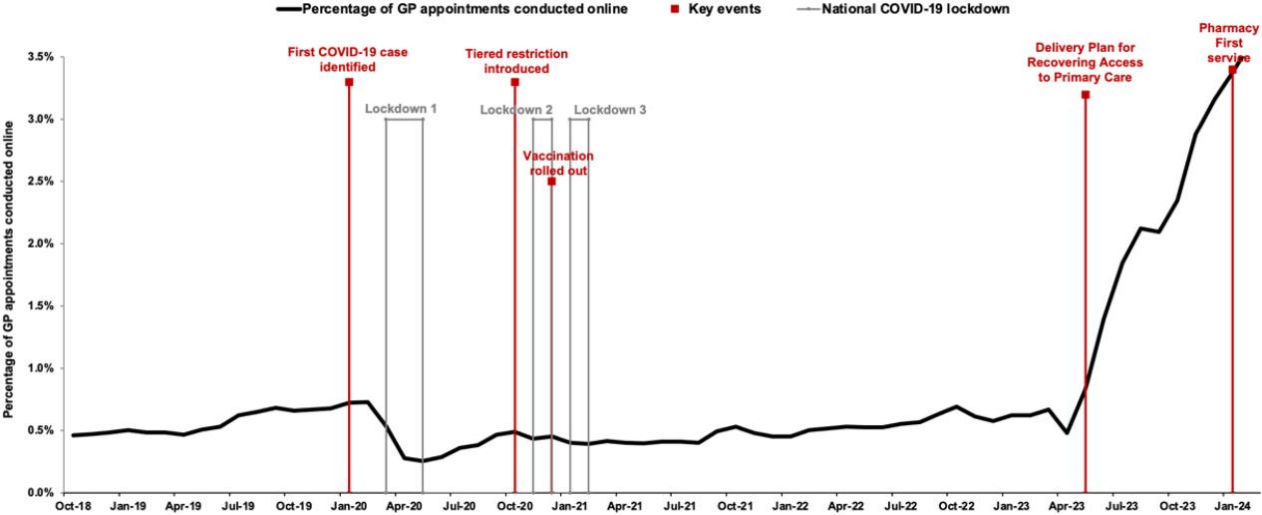
Timeline showing key events in the delivery of online/remote access to healthcare in the UK through the COVID-19 pandemic. This figure appears in colour in the online version of *JAC* and in black and white in the print version of *JAC*.

Prior to 2018 there was little appetite in the UK to seek medical attention online, but technology and the social norms that guide internet use have changed dramatically.^14^ The UK’s first NAP proposed to gather data on the extent of online purchasing and illegal sales of antimicrobial agents.^2^ Unfortunately, few data have been published evidencing progress in this area, whilst the updated 2024 NAP again highlights the importance of monitoring online antimicrobial prescribing with an emphasis on quality as well as quantity.^15^ The COVID-19 pandemic demanded a swift change in access to healthcare (Figure 1) whilst NHS workforce factors have further increased demand and reduced access to GP services. This has resulted in a rise in remote consultations which, coupled with a lack of community access to rapid diagnostics, has compounded inappropriate antimicrobial prescribing.^16,17^ These issues may have increasingly led patients to use the internet to meet their healthcare needs. As part of the GP access recovery plan,^18^ NHS England launched the ‘Pharmacy First’ initiative in May 2023, with the goal of expanding community pharmacy services to supply prescription-only medicines (POMs) for seven conditions including acute otitis media, sore throat and uncomplicated urinary tract infection.

However, there remains concern around the ability to access antibiotics through private online providers, for whom national antibiotic supply and use data, safety processes and thresholds for prescribing remain unclear.^19,20^ The context of healthcare provision around infection management, antibiotic prescribing and the use of the internet to meet healthcare needs has therefore changed significantly since our last study, whilst the necessity of improved access to meet healthcare needs requires urgent resolution. We report here an updated cross-sectional analysis of online pharmacies in 2023.

## Objectives

We aimed to improve understanding of the current state of online antibiotic sales in the UK, and specifically to (i) determine the legality of online pharmacies selling antibiotics to the UK public; (ii) describe processes for obtaining antibiotics online from within the UK; (iii) identify resulting AMS and patient safety issues; and (iv) compare data with those obtained in 2016. This will allow us to understand changes in context and set priorities for targeted research and AMS strategy going forward.

## Methods

A multidisciplinary working group (A.H.H., S.E.B., N.Z., R.A., L.W.), including healthcare professionals (HCPs) and academics with expertise in AMS agreed the study protocol, which incorporated the same data collection tool as for our previously published work.^6^ One researcher (S.E.B.) completed data collection using a computer for which the cached search history was cleared.

### Choice of search engine

In post-pandemic studies reviewing how patients access healthcare and healthcare information online, Google^®^ continues to be the most popular search engine globally,^21,22^ with Yahoo^®^ remaining a favourite choice to address healthcare needs.^22^ Consistent with the method previously published,^6^ we used both to reduce the bias of how each search engine retrieves results. The Google search was completed first (25 August 2023), followed by the Yahoo search (21 September 2023). Duplicate websites were included only once.

### Choice of search term

Internet searches using simple keywords or queries is the most favoured method to review products and services online. Consistent with the method previously published,^6^ search engine queries were conducted with the search term ‘buy antibiotics online’.

### Choice of sample size

The first position in an internet search achieves more traffic than subsequent positions,^23^ with the first page of a Google search generating the majority (∼92%) of consumer traffic.^24^ Consistent with the method previously published,^6^ a sample size of 20, to include the first 10 webpages with a unique uniform resource locator (URL) address from each search engine, meeting the inclusion and exclusion criteria, was subsequently predetermined.

### Inclusion and exclusion criteria

As in our previous study,^6^ websites were included if they were English-language vendors selling antibiotics online, for human use, to consumers within the UK. Websites were excluded if they: (i) were advertisement links; (ii) were primarily for veterinary medicine; (ii) did not ship to the UK; (iv) were exclusively designed for registered HCPs; (v) only provided delivery services for non-acute prescriptions; or (iv) had already been analysed (i.e. duplicates). Data were collected and the process for purchasing an antibiotic observed until the point of payment. Purchasing an antibiotic was defined as a payment transaction.

The first objective was to determine the legality of online pharmacies identified. Prior to 1 January 2021, the ‘distance selling logo’ had to be displayed on every webpage offering to sell POMs to the UK public.^25^ This logo was a mandatory EU requirement, but since January 2021, MHRA registration and display of the logo is no longer required in England, Wales or Scotland.^25^ Due to the Northern Ireland Protocol and EU requirements, registration with the MHRA and display of the logo is still required for online retailers operating in this region.^26^ Pharmacies based in England, Wales or Scotland must be registered with the GPhC and display of the GPhC logo was recorded. All websites were studied to identify their operating location. Evidence of GPhC registration was cross-referenced with the online register as display of the GPhC logo is voluntary. If the pharmacy was operating from Northern Ireland, evidence of the EU common logo and being on the MHRA register was also sought.

The second objective was to analyse and describe the processes for obtaining an antibiotic online from within the UK. Data were collected on prescription requirements and whether information for safe prescribing (allergies, comorbidities, pregnancy) were gathered during the assessment prior to payment transaction. Further data were collected on whether there was any verbal or non-verbal contact with an HCP prior to payment, and whether a health questionnaire was used and what format this took (‘dropdown box’ versus ‘free text’). Initial decisions regarding choice of antibiotic were defined as being ‘prescriber-driven’ or ‘consumer-driven’. As previously described,^6^ a ‘prescriber-driven’ process was when the consumer was directed through an online consultation after clicking on a specific ailment, and antibiotic selection was performed by the prescriber. A ‘consumer-driven’ process was when the consumer initiated the antibiotic purchase by selecting the antibiotic of their choice, followed by online consultation. Data were also collected on whether any safety information on adverse effects was provided to patients, whether oral or IV antibiotics were available for purchase, the standard delivery time (days) and whether an express option was available (days).

The third and fourth objectives were to identify any resulting AMS or patient safety issues by assimilation of the above findings in addition to comparing the data with those published in 2016.

After completion of data collection, any vendor identified as illegally selling antibiotics to patients within the UK were reported to the MHRA. Ethics approval was not required for this study of open-source data.

## Results

**Figure 2.**
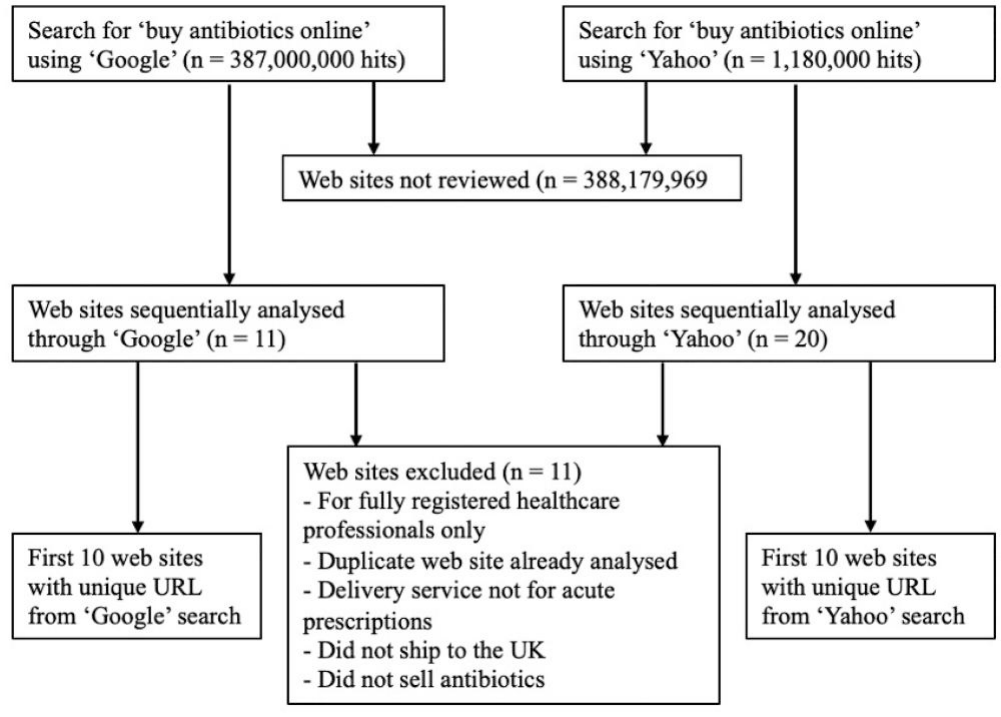
Flow diagram showing process and results from search performed on 25 August 2023 (Google) and 21 September 2023 (Yahoo).

Results of the searches performed are shown in Figure 2. Thirty-one websites were reviewed. Of the websites analysed in detail (*n* = 20), 20 (100%) websites showed evidence of operating from the UK and all had evidence of appropriate registration with the GPhC. All were non-NHS providers. Table 1 shows a comparison between 2016 and 2023 of the registration and operating locations of online pharmacies.

**Table 1.** Comparison of online pharmacies selling antibiotics to consumers within the UK from 2016 to 2023

Characteristic	Number of online pharmacies (*N* = 20)	
	2016	2023
Registered with GPhC, *n* (%)		
Yes	5 (25)	20 (100)
No	15 (75)	0 (0)
Location operating from, *n* (%)		
United Kingdom of Great Britain and Northern Ireland	5 (25)^a^	20 (100)
Unclear	10 (50)	0 (0)
India	3 (15)	0 (0)
Cyprus	2 (10)	0 (0)

^a^All those operating from within the UK in 2016 were registered with both the MHRA and GPhC in accordance with legislation at the time.^6^

**Figure 3.**
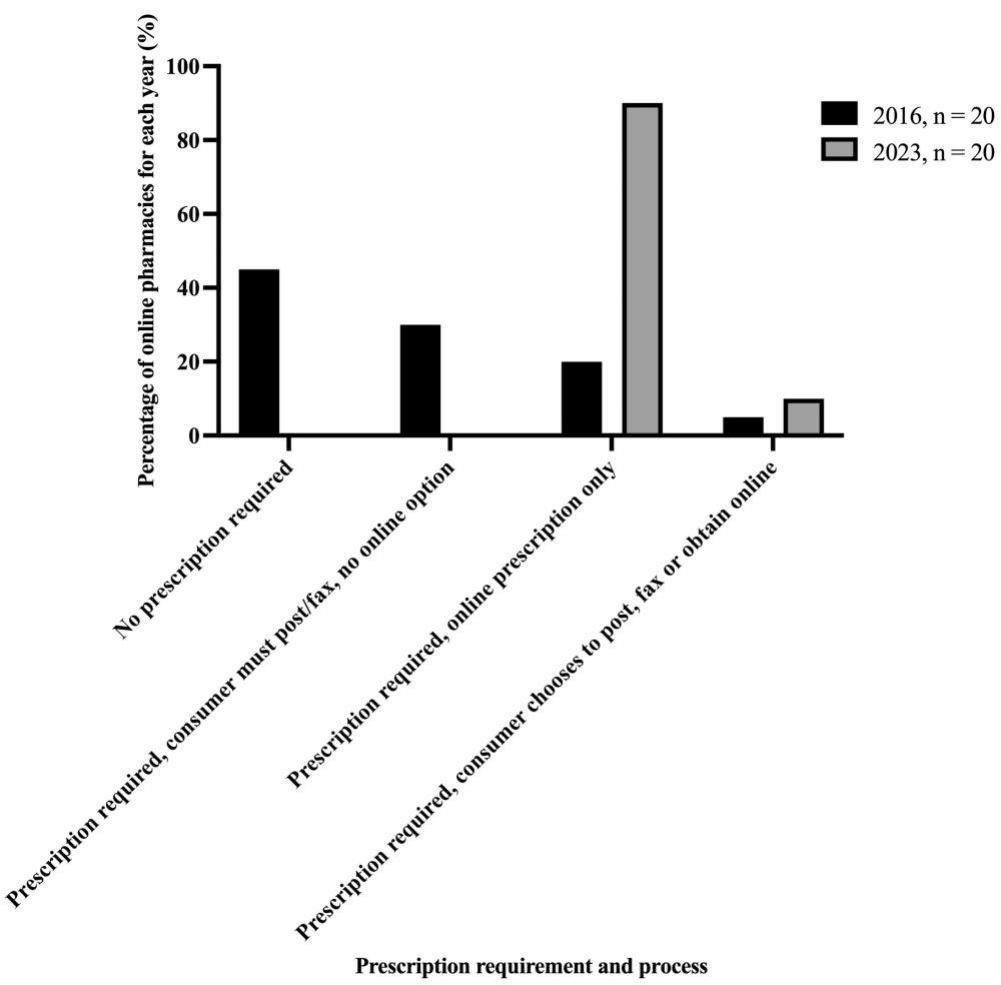
Prescription requirement and processes for obtaining an antibiotic among sampled pharmacies in 2016 and 2023.

**Figure 4.**
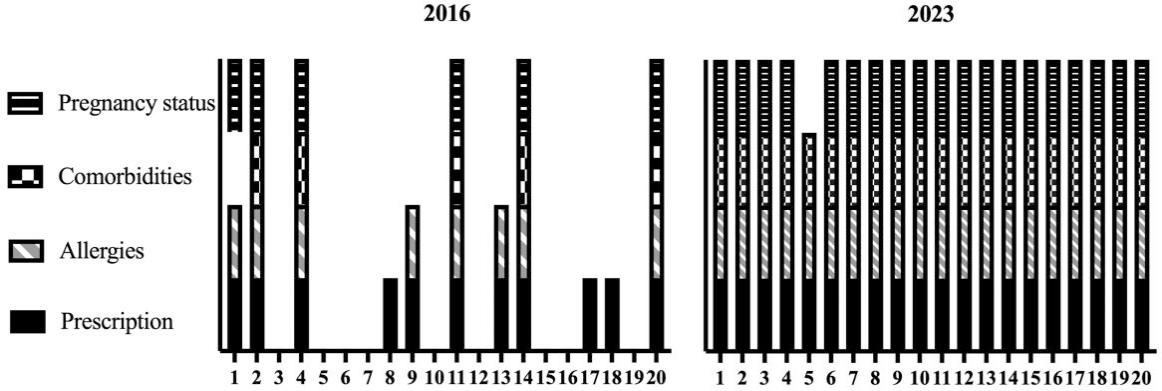
Prescription and information requirements among the top 20 online pharmacies analysed in 2016 and 2023.

Figure 3 summarizes the prescription requirements and processes for providing a prescription to the online vendor prior to purchase. All 20 UK-based online pharmacies required a prescription before an antibiotic would be dispensed, with most (*n* = 18; 90%) only offering to dispense antibiotics when the prescription was obtained through their online service. Two online pharmacies offered an additional option for dispensing the antibiotic if the user provided a private prescription. Despite all being appropriately registered, for most (*n* = 11; 55%) online pharmacies in 2023, decisions were consumer-driven for antibiotic choice, although not for dose or duration. This contrasts with 2016 when, for the vast majority (*n* = 16; 80%) of online pharmacies analysed, decisions were consumer-driven for antibiotic choice, dose and quantity.^6^ Online health questionnaires were used more frequently in 2023 (*n* = 20; 100%) in comparison with 2016 (*n* = 6; 30%) although these were inconsistent between pharmacies with varying format. Some provided ‘dropdown’ boxes for ‘yes’ or ‘no’ answers to health questions, whereas others had open questions with ‘free text’ boxes. Figure 4 correlates the requirement for prescription through each individual online pharmacy with the information that was requested, prior to antibiotic purchase, and compares the data with those collected in 2016. Improvements in prescription requirements, asking for relevant information prior to payment and dispensing can be seen, although few (*n* = 2; 10%) pharmacies analysed in 2023 offered verbal contact with an HCP, on request, prior to payment. One online pharmacy explicitly stated that they would refund money if the GP did not agree the antibiotic was necessary. All pharmacies offered oral antibiotics and none of the analysed pharmacies offered IV antibiotics. Standard delivery time to the UK varied from 1 to 5 days (mean 1.45; median 1; IQR 1–1.5). An express option was available on most (*n* = 16; 80%) websites, including one that offered the option for same-day collection from a local pharmacy.

## Discussion

This study highlights improvements made in the past 7 years regarding the availability of antibiotics online to the UK public, whilst critically highlighting several issues regarding AMS and patient safety, which should be taken forward in policy and research.

### Assessing the legality of online pharmacies

Prescribing by HCPs, all practices conducted within registered pharmacies, and advertisements for POMs are closely monitored and regulated within the UK. GPhC-commissioned research in 2022 demonstrated a rapidly shifting landscape with 1 in 4 adults likely to use online pharmacies in the future.^27^ The majority of cases involving death or harm linked to online prescribing services were associated with non-UK providers, and regulators such as the GPhC and the Care Quality Commission were unable to act because of regulatory gaps and legislative barriers.^27^ Since 2019, the GPhC has conducted inspections of online pharmacies, reporting in 2022 that only 72% met all standards, leading to enforcement actions such as condition notices and improvement notices.^28^ We did not identify any non-UK providers, which may have been influenced by these enforcement actions.

Whilst all websites analysed in 2023 were found to be appropriately registered, display of the GPhC logo was variable, consistent with its nature as a voluntary scheme applicable only to pharmacies registered in Great Britain (GB). In contrast, the EU common logo, introduced in 2015, was previously mandated for all online pharmacies in the UK. Its absence in GB since exiting the EU represents a risk as it has not yet been replaced by a logo that allows consumers to easily verify legality. This study highlights a major gap and raises concern that consumers are not currently able to reliably, consistently and rapidly identify legitimate pharmacy platforms before personal data or payment card details are entered.

### Processes for obtaining antibiotics online from within the UK

Although less than in our previous study,^6^ and as shown in Table 2, variation remains regarding the information sought via health questionnaires, and the methods used to collect this information (‘dropdown’ box versus ‘free text’), in addition to other safety procedures prior to the point of payment. A systematic review of online pharmacies reported use of a questionnaire between 10% and 81%, depending on the study.^29^ The lack of consistency is a focus of our future work. Those accessing online pharmacies are likely to have high levels of digital literacy, but there remains the need for an assessment of websites to ensure standards are met for user-friendly platforms, readability and information security, with performance data publicly available so consumers can assess these before making a choice between providers. We previously recommended mandatory implementation of a consistent and thorough health questionnaire,^6^ optimized in line with national AMS training standards for the NHS (e.g. GP TARGET toolkit). This would facilitate safe and effective prescribing whilst ensuring safety-netting and follow-up. The GMC has produced updated guidance to provide high-level principles for good practice in remote (including online) consultations and prescribing across the board, not just for infection management and AMS.^10^ This guidance should be built upon with specific recommendations embedded for AMS.

**Table 2.** Processes for obtaining an antibiotic online from within the UK in 2023

	Number of online pharmacies	
Characteristic	2016 (*N* = 20)	2023 (*N* = 20)
Consumer-driven versus prescriber-driven antibiotic choice, *n* (%)		
Consumer-driven choice of drug only, *n* (%)	0 (0)	11 (55)
Consumer-driven choice of drug, dose and quantity, *n* (%)	16 (80)	0 (0)
Prescriber-driven choice of drug, dose and quantity, *n* (%)	4 (20)	9 (45)
Online health questionnaire used to gather patient information, *n* (%)		
Yes	6 (30)	20 (100)^a^
No	14 (70)	0 (0)
Safety information provided on contraindications and side effects prior to purchasing, *n* (%)		
Yes	14 (70)	20 (100)
No	6 (30)	0 (0)

^a^Two of the websites reviewed used an online questionnaire that asked for free-text responses, whilst 18 websites used a questionnaire that allowed the user to select prefilled responses.

In this study it was not clear whether there would be feedback from the majority (*n* = 18; 90%) of online pharmacies if a discrepancy was identified between the consumer-selected antibiotic and what the prescriber deemed to be appropriate or necessary. It was also not clear on the mechanisms for safety-netting or in-person reviews. The development of suitable training tools (as above) that are specific for online providers should be actioned as a priority. These should include examples of syndromes for which online prescribing of antibiotics has been considered appropriate by key stakeholders with clear guidance on how to assess, dispense and safety-net these consultations. In addition, health professionals prescribing antibiotics online should be required to become ‘antibiotic guardians,’ complete relevant training, and demonstrate competency in line with the national framework,^30^ with decision processes for dispensing antibiotics underpinned by patient-centred, prescriber-driven rationale.

We have been unable to ascertain the volume of antibiotics dispensed through these online routes. Therefore, these data should be mandatory to report through national channels in line with antibiotics dispensed in primary and secondary NHS care. Although outside the scope of this research, centralized prescribing data are likely to become increasingly important for other non-NHS providers. This will ensure we can better understand the potential contribution to AMR and set targets for improvement.

### Additional issues for online patient safety and AMS

Direct-to-consumer medication marketing is not permitted in the UK. Despite this, antibiotics were advertised through the search results and, although this was not a primary outcome, it is raised as an ongoing concern. Technical methods that prevent advertisements must be implemented and financial penalties should be considered for those platforms that are in breach of MHRA regulations, or who are supplying antibiotics outside national AMS guidelines.

As previously described, there is an issue with accessing GP services, which ‘Pharmacy First’ will help to redress. However, this initiative covers a relatively narrow range of conditions with limited inclusion criteria using patient group directions, potentially leaving space in the market, which may have the unintended consequence of pushing online retailers to more complex areas of infection.

Our further research is seeking to identify those individuals most likely to buy antibiotics online, whilst developing automated methods to monitor this changing landscape. A 2023 study by Almomani *et al.*^31^ highlighted that education should be targeted to those most likely to engage in purchasing medicines online. We will seek to identify the factors that influence behaviour, thus facilitating the development of appropriately targeted interventions to improve online AMS and patient safety. This would benefit the safe sale of antibiotics online, especially for those who purchase without appropriate oversight or with less access to regulated healthcare routes.^32^ Education and public awareness campaigns should continue to be ambitious to be effective, and outreach could be integrated through online pharmacy platforms. Approaches that seek to engage communities online may be helpful. Formal qualitative behavioural research methods that aim to understand individual drivers will be key to positively implementing targeted education and directed safety prompts during the online process. Highlighting the benefits and risks of accessing antibiotics online should be a key component embedded within wider public awareness campaigns for AMS.

### Strengths and limitations

Websites were identified using a representative method of how consumers search for and buy products online, and by using two search engines we identified a wider range of websites. This is the first cross-sectional analysis of online pharmacies supplying antibiotics to the UK public since we last reported data in 2017. This revisit study provides a longitudinal assessment given the consistent study design but could be improved by developing an automated method to perform periodic repeated assessment. This is a focus of our future work.

This study had limitations. For example, Google or Yahoo searches are not identical when different browsers are used for the same search, or when the same search is performed at different times. This study did not assess antibiotic availability via the ‘dark web’, which may be an important route that will require further investigation. We sought to assess the availability of antibiotics online; accessibility was not assessed. Accessibility refers to how user-friendly the platforms were and seeks to identify those most likely to engage in buying antibiotics online. As discussed, these are a focus of our future work. There are further opportunities for patient safety after a payment transaction. By not purchasing antibiotics we may have missed important safety data, prompts for ‘red flag’ symptoms that require face-to-face consultations, or AMS practices that occur later. Potential patient safety and AMS risks from antibiotics obtained online may include poor-quality medication, pressured antibiotic advertising, lack of secure data storage, payment information fraud and lack of appropriate follow-up. These variables were not assessed, and this is a limitation of the study. Finally, we did not assess the ability to ‘game the system’, i.e. by changing responses to get the desired antibiotic. This is a concern for online consultations and providers should remain vigilant to it, whilst further research should be done to understand how best to limit this approach.

### Conclusions

**Figure 5.**
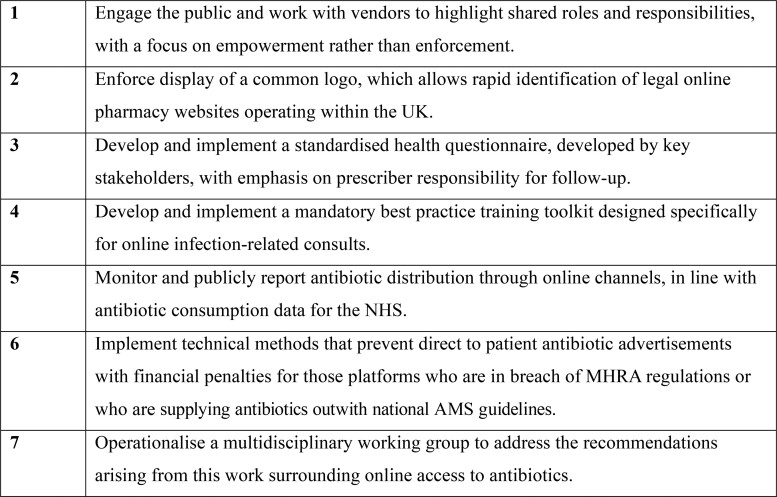
Key recommendations for lawmakers and stakeholders.

Despite progress made since our last report, important policy gaps and variability among online pharmacies has been identified in relation to the online sale of antibiotics, whilst the volume of antibiotics obtained in this way remains largely unknown. The 2024–29 UK NAP has recognized increased demand for remote and online prescribing, and this should provide focus to operationalize our key recommendations for lawmakers and stakeholders (Figure 5).

## Transparency declarations

S.E.B., N.Z., A.H.H. and R.A. are all affiliated with the National Institute for Health Research Health Protection Research Unit (NIHR HPRU) in Healthcare Associated Infections and Antimicrobial Resistance at Imperial College London in partnership with the UK Health Security Agency, in collaboration with Imperial Healthcare Partners, University of Cambridge and University of Warwick and the NIHR Imperial Patient Safety Translational Research Centre. The views expressed in this publication are those of the author(s) and not necessarily those of the NHS, the National Institute for Health Research, the Department of Health and Social Care, the UK Health Security Agency, or NHS England. S.E.B. was funded by an RCUK Innovation Fellowship through the North West MRC Scheme in Clinical Pharmacology. She received research support from Roche Pharma, educational support from Advanz Pharma, and has received consultancy and/or speaker fees from Sumitovant, Shionogi, the UK Clinical Pharmacy Association and bioMérieux. A.H.H. co-supervised a PhD part funded by Shionogi in an industrial partnership doctoral training programme, and has received consultation fees from bioMérieux. N.Z., R.S., L.W. and R.A. have no potential conflicts of interest to declare.
